# Fast-scanning small-angle X-ray scattering of hydrated biological cells

**DOI:** 10.1107/S1600577526002018

**Published:** 2026-03-30

**Authors:** Boram Yu, Mangalika Sinha, Rita Mendes Da Silva, Ulrike Rölleke, Manfred Burghammer, Sarah Köster

**Affiliations:** a Institute for X-ray Physics University of Göttingen Friedrich-Hund-Platz 1 37077Göttingen Germany; b ESRF – The European Synchrotron, 71 Avenue des Martyrs, 38043Grenoble Cedex 9, France; University of Malaga, Spain

**Keywords:** X-ray imaging, scanning SAXS, biological cells, orientation analysis, radiation damage

## Abstract

Due to their high penetration depth, X-rays enable us to obtain information from the interior of whole unsliced cells. Scanning small-angle X-ray scattering (SAXS), in particular, reveals real-space images in dark-field representation as well as structural information in reciprocal space. However, obtaining information on anisotropy and orientation from cells in an aqueous, close-to-physiological environment remains challenging. Here, we extend the recently introduced fast-scanning SAXS mode with short exposure times of a few milliseconds to such hydrated samples by combining a newly developed X-ray compatible microfluidic chamber and innovative data analysis that includes an effective noise-filtering method. This strategy enables the systematic analysis of radiation damage by quantifying the SAXS signal. Our results demonstrate that scanning SAXS can be used to obtain intracellular information of fixed-hydrated cells and the approach may in the future be applicable to living cells as well.

## Abstract 2

Fast-scanning small-angle X-ray scattering on biological cells in aqueous environments reveals intracellular anisotropy and orientation, and allows for systematic assessment of radiation damage caused by the measurements.

## 1. Introduction

The mechanics, dynamics and function of biological cells are to a great extent determined by intracellular structures, and imaging these nanometer- and micrometer-sized objects in a highly resolved manner has therefore become an essential approach for cell biology. The best-established methods for imaging of biological cells are electron and fluorescence microscopy, both of which have their distinct strengths. Electron microscopy, which comprises scanning and transmission electron microscopy, enables high spatial resolution due to the small wavelength of the electrons, albeit only of static samples. Fluorescence microscopy can be applied to samples in aqueous environments and even living cells. Super-resolution methods, such as MINFLUX, now achieve nanometer resolution (Balzarotti *et al.*, 2017 ▸; Schmidt *et al.*, 2021 ▸).

A third complementary probe for imaging is X-rays (Larabell & Nugent, 2010 ▸; Hémonnot & Köster, 2017 ▸; Weinhardt *et al.*, 2019 ▸). Due to the small wavelength, and thus inherently high spatial resolution, and the high penetration depth, whole unsliced cells can be imaged at high resolution. The electron density is imaged directly, thus avoiding the need for specific labels. Among a large variety of different X-ray imaging techniques, scanning small-angle X-ray scattering (SAXS) with a focused beam provides real-space information at moderate resolution, as well as reciprocal-space information at high resolution (Fratzl *et al.*, 1997 ▸). Therefore, this method is employed to obtain multi-scale information of hierarchical structures of biological samples (Fratzl, 2002 ▸; Weinhausen *et al.*, 2012 ▸). When first introduced, scanning SAXS was employed to investigate comparatively radiation-stable biological materials, such as wood (Fratzl *et al.*, 1997 ▸), bone (Rinnerthaler *et al.*, 1999 ▸) and teeth (Tesch *et al.*, 2001 ▸). More recently, the authors and others have also applied it to radiation-sensitive biological cells and tissues (Weinhausen *et al.*, 2012 ▸; Müller *et al.*, 2010 ▸; Bernhardt *et al.*, 2016 ▸, 2017 ▸; Reichardt *et al.*, 2020 ▸). Such studies are challenging, as the electron-density contrast between the cells and the surrounding aqueous solution is weak and the signal easily gets lost due to radiation damage.

In the past, cells were studied in freeze-dried (Weinhausen *et al.*, 2012 ▸; Wilke *et al.*, 2012 ▸; Bernhardt *et al.*, 2016 ▸, 2017 ▸), frozen-hydrated (Priebe *et al.*, 2014 ▸), fixed-hydrated and even living conditions (Weinhausen *et al.*, 2014 ▸; Priebe *et al.*, 2014 ▸; Bernhardt *et al.*, 2017 ▸). Typically, the total scan time for these studies extends from ∼15 min to over one hour per single cell, depending on scan step sizes and exposure time. More recently, thanks to the high photon flux and consequently shorter exposure times after the EBS (Extremely Brilliant Source) upgrade at ESRF (The European Synchrotron), fast continuous scanning has enabled us to collect data from several hundreds of freeze-dried cells during a total experiment time of ∼10 h (Cassini *et al.*, 2020 ▸). Dark-field contrast images could be analyzed, including semi-automated cell segmentation of a large number of cells and *q*-dependent intensity profiles.

So far, fast-scanning SAXS has been constrained to freeze-dried samples, because the low signal-to-noise ratio (SNR) associated with the short exposure times is even lower in hydrated samples. In earlier studies employing conventional slower scanning, data obtained from fixed-hydrated or living cell samples enabled the analysis of dark-field contrast images and *I*(*q*) profiles (Weinhausen *et al.*, 2014 ▸), and spatial structural information such as the extent of anisotropy (Bernhardt *et al.*, 2017 ▸). However, derivation of meaningful structural information from fast-scanning SAXS measurements on hydrated samples remains limited by low SNR. One challenge lies in adjusting the thickness of the water layer that surrounds the cells. It needs to be thick enough to fully enclose the mammalian cells and at the same time as thin as possible as water leads to absorption and secondary scattering of the inherently weak scattering signal stemming from the cells. Previous microfluidic chambers (Köster & Pfohl, 2012 ▸; Ghazal * et al.*, 2016 ▸; Levenstein *et al.*, 2025 ▸) were used to obtain dark-field images from hydrated cells using long exposure times (Weinhausen & Köster, 2013 ▸), but local orientation of alignment of cellular components as measured by SAXS was not detectable unless the cells were freeze-dried (Weinhausen *et al.*, 2012 ▸; Hémonnot *et al.*, 2016*a* ▸).

Here, we present a strategy for imaging intracellular structures in fixed-hydrated samples by fast-scanning SAXS including (i) a novel design for an X-ray compatible microfluidic chamber featuring continuous buffer flow and a minimized water-layer thickness, and (ii) an effective noise-filtering method to extract meaningful information. We demonstrate that structural information, such as local orientation and anisotropy, can be obtained and we systematically assess the influence of radiation damage on the SAXS signal. Thus, our approach provides suitable measurement parameters for fast-scanning SAXS on biological cells in aqueous environments.

## 2. Materials and methods

### 2.1. Sample preparation

We use SW-13 cells, derived from adrenocortical carcinoma (Leibovitz, 1973 ▸), stably transfected with fluorescence keratin hybrid expression (HK8-CFP, HK18-YFP) (Strnad *et al.*, 2002 ▸; Windoffer *et al.*, 2004 ▸). These cells are cultured in high glucose (4.5 gL^−1^) Dulbecco’s Modified Eagle’s Medium (DMEM, D6429; Merck KGaA, Darmstadt, Germany) supplemented with 10%(*v*/*v*) fetal bovine serum (FBS, 10270-106, Gibco), and 1%(*v*/*v*) 100 UmL^−1^ penicillin and 0.1 gL^−1^ streptomycin (pen-strep, 15140122; Gibco). Following the thawing process, the cells are incubated at 37°C in a water-saturated atmosphere with 5% CO_2_.

Silicon nitride membranes (Si_3_N_4_, hereafter denoted by SiN, with a frame size of 10 mm × 10 mm, a frame thickness of 200 µm, a window size of 1.5 mm × 1.5 mm, and a window thickness of 1 µm; Silson Ltd, Warwickshire, UK) are plasma activated at 50 W for 40 s (Zepto, Diener electronic GmbH & Co. KG, Ebhausen, Germany) to render the surface hydrophilic. Subsequent to the plasma activation, the membranes are immersed in ultrapure water and undergo UV treatment for 30 min for sterilization. The sterilized membranes are then positioned on a 12-well cell-culture plate, with the flat side oriented upwards. A solution of 40 µL of 5%(*v*/*v*) fibronectin (F1141, Merck KGaA) in phosphate-buffered saline (PBS, D8537, Merck KGaA) is applied to the membrane to enhance cell adhesion and incubated at 37°C for one hour.

Once ∼80% confluency of the cultured cell layer is reached, the cells are detached by adding 0.25%(*v*/*v*) trypsin with 0.02%(*w*/*v*) EDTA (P10-020500, PAN-Biotech GmbH, Aidenbach, Germany) in PBS, and thereafter suspended in fresh culturing medium. Following this step, the cells are seeded onto the membrane at an initial concentration of 1.5 × 10^5^ cells ml^−1^, or 6.25 × 10^5^ cells cm^−2^, *i.e.* 1.5 ml in each well in a 12-well cell-culture plate. After a 24-hour incubation, the membranes are washed with PBS and fixed for 15 min with a 4% methanol-free formaldehyde solution (28906, Thermo Fisher Scientific Inc., Darmstadt, Germany) in PBS, followed by three rinses with PBS (Thavarajah *et al.*, 2012 ▸). This procedure leads to ‘fixed-hydrated’ samples. Here ‘fixed’ refers to chemical fixation described above that is commonly employed in cell biology to preserve native protein structures for further sample preparation and imaging. ‘Hydrated’ refers to the fact that the cells are thereafter kept in aqueous environments, *i.e.* suspended in buffer, rather than freeze drying the cells. This procedure leads to cell samples, where the protein structures are to a great extent preserved in their native state; however, the cells are not alive anymore and thus much easier to handle both in terms of measurements and in terms of safety regulations at the synchrotron. We are aware that the chemical fixation process, although very established and common in the cell biology community, does induce alterations to the nanoscale structures of the cell, as we have previously shown by comparing chemically fixed and living cells using an otherwise identical setup (Weinhausen *et al.*, 2014 ▸).

Visible-light phase contrast and fluorescence images of the membranes with fixed cells are recorded using an inverted microscope (IX83 Olympus, Hamburg, Germany), equipped with a UCPlanFLN 20× objective (0.7 NA, Olympus) and UPLFLN 40× objective (0.75 NA, Olympus). For fluorescence imaging, an IX3-FGFPXL filter set (excitation filter: 460–480 nm; emission filter: 495–540 nm; dichroic mirror: 490 nm; Olympus) is employed. Thereafter, the fluorescence images are sharpened by Fourier filtering (Petrou & Petrou, 2010 ▸) using the Python tool box *scikit-image* (Van der Walt *et al.*, 2014 ▸). The membranes are individually stored in PBS with 1%(*v*/*v*) pen-strep and transported to ESRF.

### 2.2. The microfluidic chamber

Master wafers for the polydimethylsiloxane (PDMS, Sylgard 184, Dow Corning, Midland, MI, USA) structures including the flow channels are fabricated by standard photolithography. Briefly, SU-8 negative photoresist (SU-8 3050, MicroChem, Newton, MA, USA) is spin coated onto a silicon wafer (2-inch, MicroChemicals, Ulm, Germany) to a height of 210 µm. To reach this height, the process has to be repeated twice. The resist is exposed to UV light through a mask containing the structure [see Fig. S1(*a*) of the supporting information] and developed. The wafers are coated by fluorosilane (1H,1H,2H,2H-perfluorooctyltriethoxysilane, 97%, AB 104055; abcr GmbH, Karlsruhe, Germany) and PDMS replicas are produced from the wafers. One PDMS replica needs to be thick enough (10 mm) to distribute the pressure generated by clamping the edges with the metal frame to the SiN membranes, thus providing a good seal. The second replica is produced as thin as possible (5 mm) so as to position the sample as close to the X-ray aperture as possible. After curing, the PDMS is removed from the wafer, and holes are punched for the inlet, outlet (diameter 0.75 mm) and X-ray observation window (diameter 3.5 mm) [see Fig. S1(*b*)]. A schematic drawing of the chamber preparation is shown in Figs. S2(*a*)–S2(*d*). Just before the experiment, the PDMS replicas are plasma activated at 15 W for 60–80 s (Tergeo, PIE Scientific LLC, Union City, CA, USA). The SiN membrane with the cells is rinsed with ultrapure water to remove salt and to avoid crystal formation on the outer side of the membrane during scanning. As the cells are not alive anymore after the fixation, we expect no influence of the short exposure to ultrapure water on intracellular structures. The water on the side opposite of the cells is completely blotted off with filter paper. The SiN membranes are placed above the observation windows in the plasma-activated PDMS replicas, and the two halves are assembled under a stereomicroscope (Wild M3Z, Leica Microsystems GmbH, Wetzlar, Germany). Tubing (inner diameter: 0.38 mm; outer diameter: 1.09 mm; polyethylene; Becton, Dickinson and Company, NJ, USA) filled with degassed PBS is inserted into the chamber, and all components are clamped by the metal frames. The assembly process, including the sample preparation process explained in Section 2.1, is illustrated in Figs. S2(*e*)–S2(*j*).

**Figure 1:**
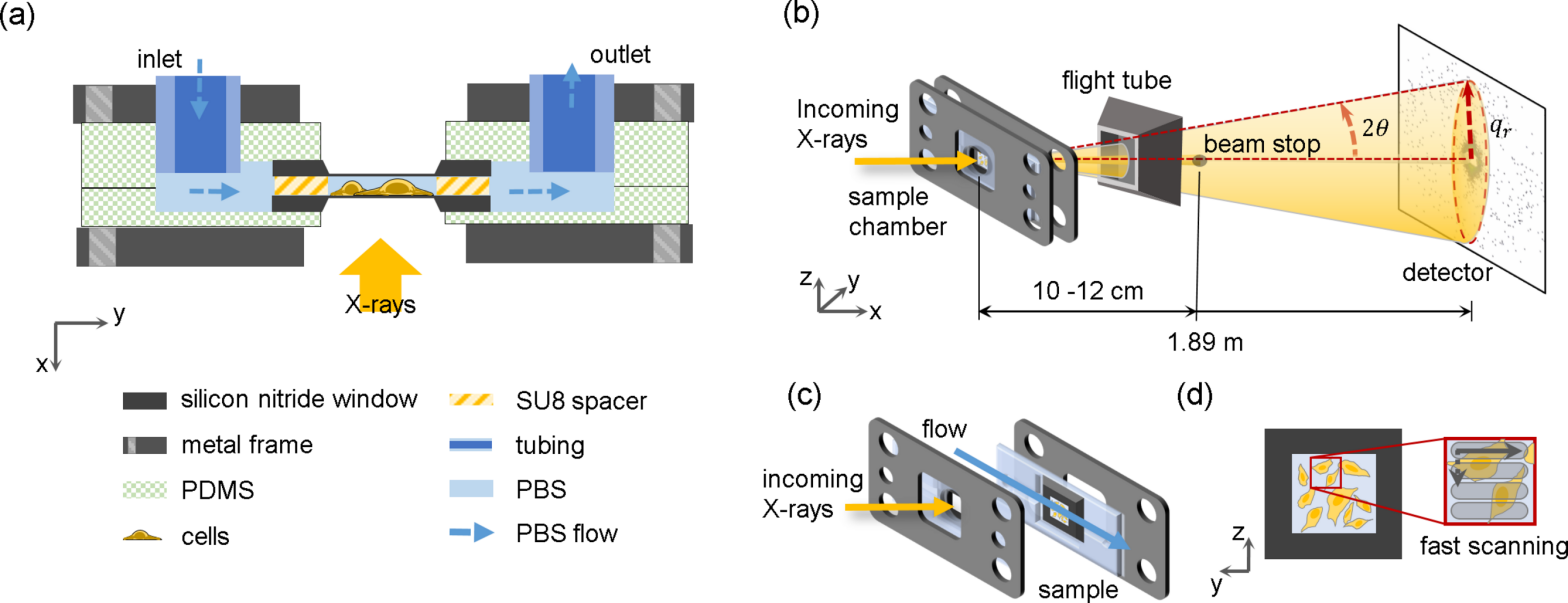
Experimental setup. (*a*) Schematic side view of the dedicated flow chamber (see legend for the individual components). Two SiN membranes are separated by 20 µm using SU-8 spacers. This ‘sandwich’ is enclosed by PDMS layers and clamped by metal frames. The buffer flow is represented by blue dashed arrows. (*b*) Schematic drawing of the SAXS experiment (not to scale). The sample (fixed-hydrated cells on a SiN membrane) is scanned through the focused X-rays. The beamstop blocks the direct beam, and the two-dimensional detector records the scattered X-rays. The red dashed lines represent the scattering angle 2θ between the incoming beam direction and the scattered X-rays. (*c*) The flow within the microfluidic chamber is oriented horizontally in perpendicular direction to the incoming beam, thus along the *y* axis. (*d*) Representation of the scanning scheme; the SiN membrane is moved along the *y* and *z* axes to obtain a raster scan of the selected region with the specified step numbers and sizes.

When fully assembled, the sample chamber is composed of two SiN membranes, one of which the cells are grown, and the other one with an SU-8 spacer of a thickness of 20 µm (Silson Ltd) on its flat side [see Fig. 1 ▸(*a*) and Fig. S1(*c*) for more details], thereby enabling liquid flow over the window region. The two SiN membranes are arranged in such a manner that their flat sides face each other. The sandwiched membranes are encased by two pieces of PDMS with a flow channel structure of 1.5 mm width and 30 mm length, including a 10 mm square positioned centrally, wherein the membranes are placed [see Fig. S1(*a*)]. The components are clamped by two aluminium frames [see Fig. S3(*a*)], which ensure leak tightness during prolonged operation. The assembled chamber is inserted in a dedicated chamber holder compatible with the X-ray sample stage described in Section 2.3 [details are provided in Figs. S3(*b*) and S3(*c*)].

The resulting chamber has an operation time of up to 24 h and can in principle be used with flow rates of more than 1000 µL h^−1^. A syringe pump (base and single module, Cetoni GmbH, Korbussen, Germany) is used to control the volume flow and we adjust the flow rate between 20 µL h^−1^, which corresponds to 0.18 mm s^−1^, and 1000 µL h^−1^, which corresponds to 9.26 mm s^−1^, for the given channel geometry. When operating the chamber without flow, we observe bubble formation during the scanning, which prohibits further measurements.

### 2.3. Scanning SAXS

The scanning SAXS experiments are performed at the nano-branch of beamline ID13 of the European Synchrotron (ESRF) in Grenoble, France. A sketch of the experiment geometry is shown in Fig. 1 ▸(*b*). The X-ray beam from the undulator is monochromated by a Si 111 channel-cut monochromator to an energy of 15 keV. The beam is focused through multiple steps. The beam is first pre-focused by beryllium parabolic refractive lenses. It is then focused by multilayer Laue lenses (MLLs) with a focal distance of ∼60 mm to a final size of 250 nm × 250 nm at a flux of 1.09 × 10^12^ photons s^−1^. At the endstation, the focused beam undergoes conditioning by a square-shaped 40 µm × 40 µm PtIr aperture (order-sorting aperture, OSA) and is subsequently targeted at the sample. The distance of the OSA from the focus is 8 mm. Thus, the beam is defined by the first order of the MLL focusing optics. The OSA serves the purpose to block the higher orders. At the same time, the OSA has the function of a classical guard aperture to clean up the SAXS signal. The typical divergence of the beam is 1.33 mrad. At the downstream side of the sample, a small helium-filled flight tube is placed to reduce the air scattering and the beamstop is located right outside the exit window of the tube to block the direct beam. Consequently, the distance between the sample and the beamstop is 10–12 cm. Scattered X-rays are recorded with an Eiger X 4M two-dimensional X-ray detector (2070 × 2167 pixels, *i.e.* ∼4 megapixels with a pixel size of 75 µm × 75 µm; Dectris, Baden, Switzerland). The distance from the sample to the detector is 1.89 m, as determined by measuring AgBeh powder in the empty chamber.

The assembled sample chamber is mounted on a piezo translation stage, which is installed on a hexapod. The hexapod is used for coarse positioning, while the piezo stage facilitates finer motion with a minimum step size of 20 nm; thus, this configuration enables fast scanning, which is implemented as a continuous scanning mode in a row-by-row manner, *i.e.* no signal is recorded when the piezo is moved back to the initial position. This entails overhead time at the very beginning of each scan as well as between the scan lines, amounting to 15–20 min per scan. During this time the sample is exposed to the X-rays. The scanning is conducted along the *y* and *z* axes as shown in Fig. 1 ▸, which form a vertical plane perpendicular to the beam path. Photographs of the end station after mounting the chamber are shown in Fig. S4. The mounted sample is aligned with the beam using an on-axis microscope that can be moved in and out of the beam.

During the measurement, the step sizes for the *y* and *z* axes are selected with respect to the beam size: similar to the beam size (200 nm × 200 nm), which yields the highest spatial resolution but also higher radiation damage; four times the beam area (500 nm × 500 nm) to minimize radiation damage at the expense of resolution; or asymmetrically (200 nm × 500 nm), which balances radiation damage and spatial resolution. The exposure time is set just above the detector’s lower limit (1.34 ms) and thus long enough to collect a sufficient signal while minimizing radiation damage. The radiation dose *D* can be estimated by 
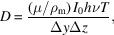
where μ/ρ_m_ = 1.60 cm^2^g^−1^ is the ratio between mass attenuation coefficient and mass density of the cellular material (Berger *et al.*, 2010 ▸), *I*_0_ is the photon flux of the incoming beam, *h*ν is the photon energy, *T* is the exposure time per scan point, and Δ*y* and Δ*z* are the step sizes (Howells *et al.*, 2009 ▸; Weinhausen *et al.*, 2012 ▸). The measurement parameters and estimated radiation doses are listed in Table 1 ▸.

In order to analyze the decay of the ordered structures in response to the radiation dose, scanning SAXS is conducted in a single area multiple times with the same scanning parameters.

### 2.4. Data analysis

Each scan consists of 40000–250000 individual scattering patterns. The scattering intensities, along with their corresponding positions, are used to calculate a dark-field contrast image, where the total scattering intensity is plotted on a color scale (Bunk *et al.*, 2009 ▸; Weinhausen *et al.*, 2012 ▸).

The background and foreground regions are segmented based on the dark-field images. To account for beam variations during the extended scans (15–20 min per scan), the background region is determined by automated cell segmentation via adaptive thresholding [*ACSAT* (Shen *et al.*, 2018 ▸)]. *ACSAT* is a cell-segmentation method that employs global and local thresholding iteratively. In each iteration, the algorithm detects the foreground regions at various threshold values and creates regions of interest (ROIs) containing a cell via a morphological operation. It then selects the minimum threshold value that leads to the maximum number of ROIs. This process is conducted for the entire image (global thresholding) first, and is then repeated in a rectangular ROI (local thresholding). During this process, any detected ROIs smaller than a minimum area 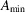 are filtered and removed. We choose 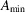 = 500 pixels for scans with 500 nm × 500 nm step size, 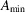 = 750 pixels for 200 nm × 500 nm and 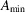 = 1000 pixels for 200 nm × 200 nm step size. The cell mask created by *ACSAT* is shown in Fig. S5(*b*). Remaining noise is filtered out using a histogram-based Gaussian filter, which removes pixels whose intensity exceeds three standard deviations (3σ) from the Gaussian distribution of the masked regions’ intensities. These filtered pixels and the cell regions are dilated by three pixels with a circular structure element, and the background region is defined as non-selected pixels. The background signal, which is used to correct the data in a row-by-row manner, is defined by averaging the scattering patterns of all pixels of the background region in a single row.

The local orientation, indicating the average orientation of all structures at the measurement position, is obtained by calculating the circular mean and the circular variance (Allen & Johnson, 1991 ▸; MacArthur & Thornton, 1993 ▸) of the azimuthal intensity profile, which are used to determine the orientation and anisotropy, respectively. In order to calculate the azimuthal intensity profile, we make use of the point symmetry of the scattering patterns to complete the missing regions close to the beamstop. The azimuthal intensity profile is calculated using the minimum *q* (*q*_2_, see Section 3.2), which covers the missing regions after compensation, as the lower limit. From the azimuthal intensity profile, the circular mean and variance are obtained, see Fig. S6 for a graphical representation of this procedure. By plotting the orientation and anisotropy values at their corresponding positions, an orientation map as well as an anisotropy map are obtained. We here refer to the orientation of the actual cellular components in real space that is perpendicular to the orientation seen in the corresponding scattering signal. Following these analyses, the ordered intracellular structures, in our case, for example, bundles of keratin filaments, are visible, and the decay of the order with increasing radiation dose is analyzed. A detailed graphical description of the data-analysis workflow is provided in Fig. S7.

The decay of the ordered intracellular structures as a response to the radiation is computed for individual cells. For this analysis, each individual cell in the background-subtracted dark-field contrast image is annotated by marker-controlled watershed segmentation (Meyer, 1994 ▸). In order to annotate the cells individually, a distance map is calculated for a watershed segmentation. For separating adjacent cells, we make use of the fact that each cell contains one nucleus. A nuclei mask is created via morphological reconstruction. Morphological reconstruction is a feature-extraction method based on pixel connectivity that preserves the original shape of the nuclei (Vincent, 1993 ▸). Applying the mask as the local minima for the watershed segmentation, all cells, including adjacent cells, are annotated individually. The final cell masks, the nuclei masks and the cell-annotation result are shown in Figs. S5(*b*)–S5(*d*).

Following the annotation step, anisotropy distributions of ordered intracellular structures of individual cells are calculated. As ordered structures are more anisotropic, pixels in a cytoplasmic region with high anisotropy values are selected. The cytoplasmic region is defined as the area that is included in the cell mask but excluded from the nuclei mask. To make this selection, an anisotropy distribution of the masked background region is plotted and its 99th percentile value is used as the thresholding value. To track changes in the anisotropy distributions induced by radiation, the distributions are depicted as violin plots against scan number. The difference in mean value between the first and the second distribution is set as the anisotropy decay caused by radiation damage and then compared against different radiation doses. All data analysis is performed using MATLAB R2020a (The MathWorks, Inc., Natick, MA, USA) scripts, including the *Image Processing Toolbox* and functions of the *Nanodiffraction Toolbox* published by Nicolas *et al.* (2017 ▸).

## 3. Results and discussion

We perform fast-scanning SAXS in biological cells grown on SiN membranes and incorporated in a dedicated microfluidic flow chamber. Due to the short exposure time of a few milliseconds and the water layer surrounding the cells, as well as the low electron-density contrast between water and cells, the collected SAXS data show a low SNR, making the data analysis challenging (Bernhardt *et al.*, 2017 ▸; Nicolas *et al.*, 2019*b* ▸; Reichardt *et al.*, 2020 ▸). Here, we deliberately apply our method to fixed-hydrated cells in order to prepare for future measurements on living cells. The challenge is, however, that the contrast between the aqueous environment and the cellular material is lower than between air and dried cell samples. We propose a novel approach to filter noise in the data that involves constraining the *q* range based on a systematic analysis of dark-field contrast images calculated from different *q* ranges. This method allows for the extraction of structural information of intracellular components and we demonstrate its utility by quantitatively analyzing the impact of radiation damage on intracellular structures.

### 3.1. Optimization of the *q* range for data analysis

In scanning SAXS, dark-field contrast images are calculated by summing up the intensity values of all pixels across the entire detector area (Fratzl *et al.*, 1997 ▸; Priebe *et al.*, 2014 ▸) or just within a circular region around the primary beam (Bunk *et al.*, 2009 ▸; Weinhausen *et al.*, 2012 ▸). In many cases, the appropriate detector range is chosen by a qualitative inspection of the resulting dark-field images. In our case, however, due to the fast scanning, short exposure times and, therefore, strong noise, such an approach is difficult. Instead, we choose an objective way to optimize the *q* range for the dark-field images, which is then used for further analysis of the SAXS data as well. We calculate the magnitudes of the scattering vectors *q* as 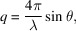
where λ is the wavelength of incoming X-rays and θ is half the scattering angle [see Fig. 1 ▸(*b*)]. For our setup, the maximum *q* value is 5.465 nm^−1^.

**Figure 2:**
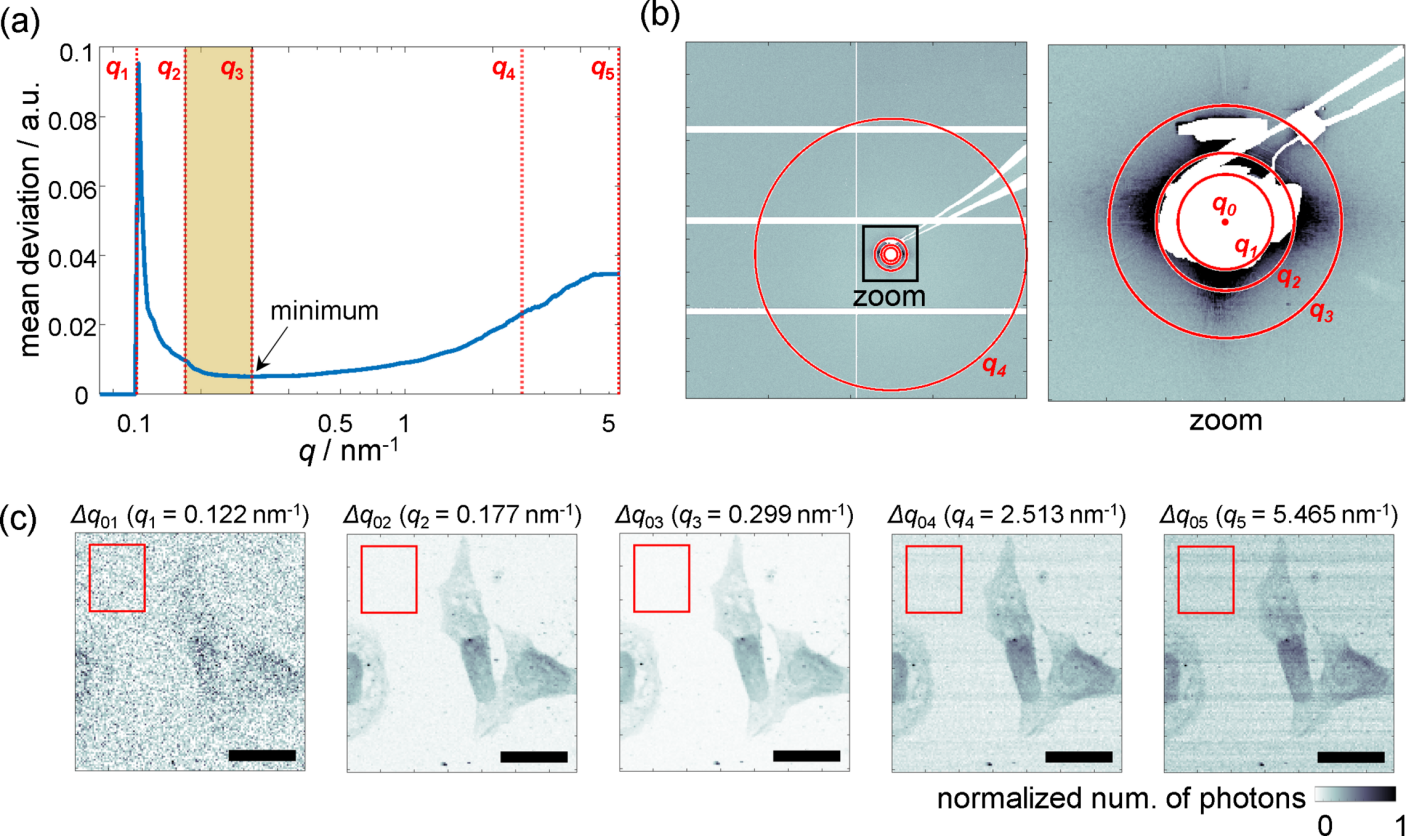
Optimization of the *q* range. (*a*) The MD of the background region [red boxes in panel (*c*)] is plotted against the magnitude of the scattering vector *q*. The vertical red dashed lines correspond to the selected *q* values in (*b*) and (*c*). The chosen *q* range (yellow), reaches from *q*_2_ to *q*_3_. (*b*) Summed scattering pattern from 40000 individual patterns of the whole detector view (left) and an enlarged view (right). The beamstop and aperture-edge artefacts are masked and shown in white. (*c*) Series of X-ray dark-field contrast images obtained with varying maximum *q* values *q*_*i*_ as shown in panel (*b*); *q*_0_ is the scattering center, *q*_1_ is the minimum radius at which scattering signals appear, *q*_2_ is a circumscribed circle of the beamstop (ignoring neighboring artefacts), *q*_3_ is the minimum of the MD curve in panel (*a*), *q*_4_ corresponds to the maximum circle within the detector area and *q*_5_ corresponds to the whole detector area. In each case the minimum *q* value used for the calculation of the dark-field image is *q*_0_. The scanning is performed with 0.5 µm × 0.5 µm step size and 5 ms of exposure. Scale bars are 20 µm.

We determine dark-field contrast images using various upper *q* values ranging from 0.003 to 5.465 nm^−1^ with 0.003 nm^−1^ intervals, resulting in 1795 data points in total. The intensity values in the images are normalized to a range of [0, 1]. We compare the noise level of the individual images, defined by the mean deviation (MD, also called the mean absolute deviation) of a rectangular featureless region of size *a* × *b*: 
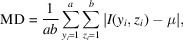
where the mean intensity is given by 
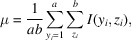
and *I*(*y*_*i*_, *z*_*i*_) is the intensity of the pixel (*y*_*i*_, *z*_*i*_) (Sari *et al.*, 2012 ▸; Rajabi & Zirak, 2016 ▸). The MD is more suitable than the standard deviation because it is less sensitive to extreme values (Gorard, 2005 ▸). The MD values for the selected background region are plotted against *q* in Fig. 2 ▸(*a*). As the MD is obtained from the normalized dark-field images, it is indicative of the SNR of the image. The scattering center *q*_0_ is chosen as the lower bound of the optimized *q* range and the minimum of the MD curve, *q*_3_, is chosen as the upper bound, because here the scattering signal from the cells is sufficiently strong while the background noise is minimal. Before determining the minimum of the MD curve, it is smoothed with a window size of 38 data points, determined by the elbow method (Thorndike, 1953 ▸). The choice of the upper bound is illustrated by an image series for selected *q* values shown in Fig. 2 ▸(*c*). Here, we show five images for comparison, which are selected as described in the caption. The cell contours are hardly visible when the upper *q* value is very small. For *q*_2_ = 0.177 nm^−1^, the cell contours are clearly visible; however, the *q* range is still very small [see Fig. 2 ▸(*b*)]. This indicates that most signal stemming from the cells is concentrated close to the center of the scattering pattern at small *q* values. When *q*_3_ = 0.299 nm^−1^, corresponding to the minimum of the curve, the background area surrounding the cells becomes brighter than in the second image, indicating improved contrast. However, upon expanding the calculation region to *q*_4_ = 2.513 nm^−1^ and *q*_5_ = 5.465 nm^−1^, stripes along the fast-scanning direction [*y* axis in Fig. 1 ▸(*d*)] appear. These stripes are considered additive noise (see Fig. S8) because they are independent of the original signal. This suggests that the observed noise is caused by exterior sources, such as mechanical vibrations of the stage during scanning. These example dark-field contrast images show that the noise level depends on the *q* range considered for the calculation. For the work presented here, we determine *q*_opt_ to be *q*_3_, as the optimized upper bound for the *q* range from the specific dataset shown in Fig. 2 ▸, and use it for the analysis of all datasets.

### 3.2. Analysis of the local orientation of cellular structures

**Figure 3:**
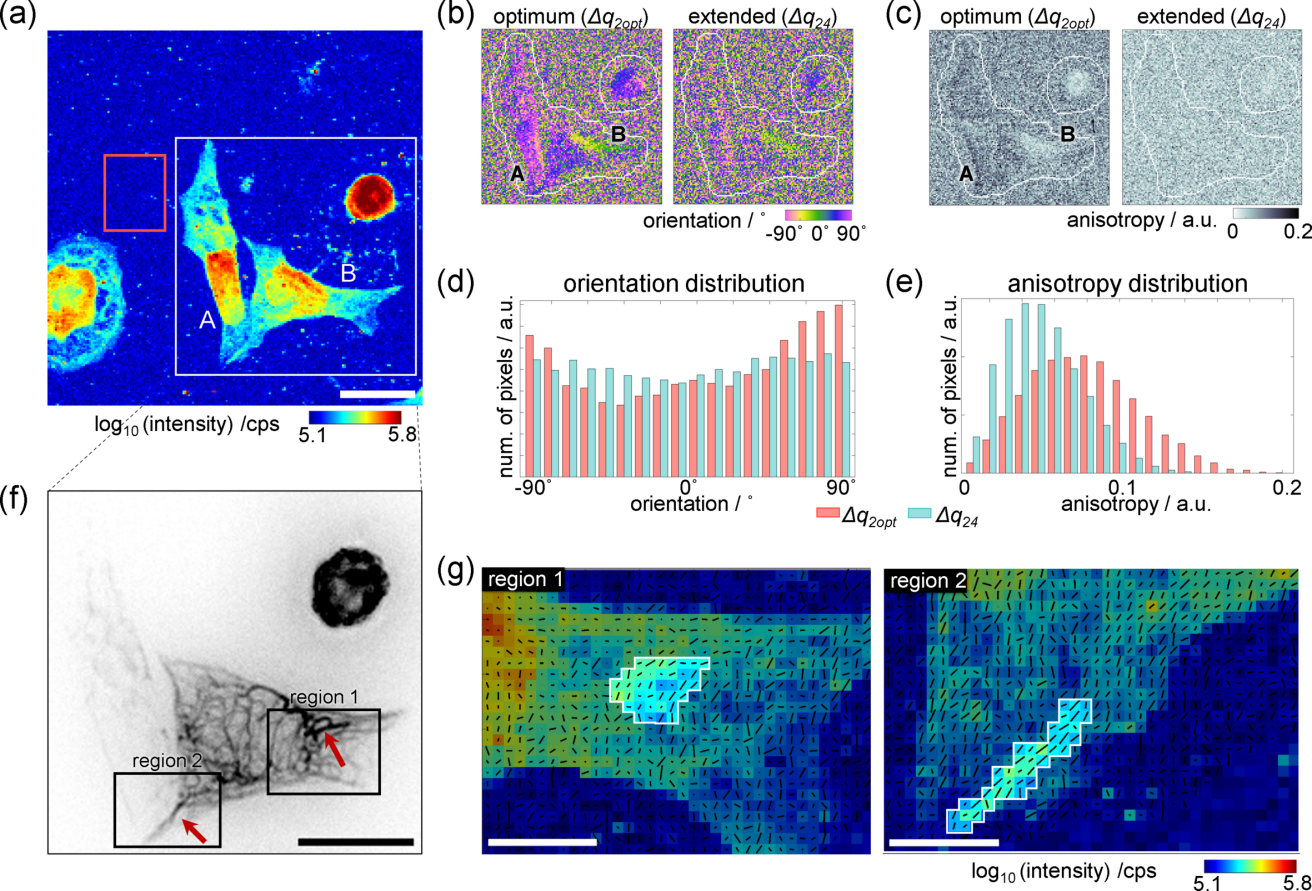
Orientation of cellular structures. (*a*) Dark-field contrast image of the same dataset as shown in Fig. 2 ▸(*c*) with a larger field of view. The red rectangular region in Fig. 2 ▸(*c*) is also added for comparison. (*b*) Orientation maps of the white rectangular region in panel (*a*), calculated using different *q* ranges: Δ*q*_2opt_ from *q*_2_ to *q*_opt_ (optimum range as discussed in Section 3.1) and Δ*q*_24_ from *q*_2_ to *q*_4_ (extended range for local orientation analysis). (*c*) Corresponding anisotropy maps. The white outlines in panels (*b*) and (*c*) delineate the border of the background area used for the background subtraction. The grayscale indicates the degree of anisotropy. A value of 0 corresponds to an azimuthal intensity profile that is equally distributed over all angles, whereas a value of 1 would correspond to an orientation strictly only in one direction. Histograms of (*d*) orientation and (*e*) anisotropy distributions of cells A and B as indicated in panels (*a*)–(*c*). (*f*) Inverted-grayscale epifluorescence image of the same cells, acquired prior to the microfluidic chamber assembly. The dark structures are distinct keratin bundles within the cell. (*g*) Dark-field contrast images of the two regions 1 and 2 within the black boxes in panel (*f*), where ordered keratin structures are visible. The black lines in each pixel represent the orientation and anisotropy by their angle and length, respectively. Scale bars in panels (*a*) and (*f*): 20 µm. Scale bars in panel (*g*): 5 µm.

For orientation analysis, we chose *q*_2_ as the lower bound of the *q* range in order to obtain the complete information in all scattering directions. Thus, using the *q* range from *q*_2_ to *q*_opt_, we further analyze the example dataset shown in Fig. 2 ▸. A dark-field contrast image of a slightly larger field of view is shown in Fig. 3 ▸(*a*), on a logarithmic color scale. Distinct intensity differences are observed between the cytoplasmic and nuclear regions, with some variations in intensity within each region. As shown previously, these variations arise from inhomogeneous distributions of the cellular components, such as the cytoskeleton in the cytoplasm (Weinhausen *et al.*, 2012 ▸), or different degrees of DNA compaction in the nuclei region (Hémonnot *et al.*, 2016*b* ▸).

Thus, the dark-field contrast images provide real-space information and inform us about the location of cellular structures such as the nucleus. However, in scanning SAXS, the scattering patterns recorded in each pixel contain additional reciprocal-space information on the local structure within the cell. In particular, we analyze the local orientation and calculate orientation and anisotropy maps, as shown in Figs. 3 ▸(*b*) and 3 ▸(*c*), respectively. Here, we restrict the analysis to the area within the white box shown in Fig. 3 ▸(*a*).

We compare the local orientation for two different *q* ranges, *i.e.* the optimum range Δ*q*_2opt_ from *q*_2_ to *q*_opt_ and the extended range Δ*q*_24_ from *q*_2_ to *q*_4_, as shown in Figs. 3 ▸(*b*) and 3 ▸(*c*). In Fig. 3 ▸(*b*), an orientation of 0° corresponds to the horizontal direction. For calculating the azimuthal intensity profile at least half the azimuth is required; here, we include the full azimuth to account for the low SNR (Bernhardt *et al.*, 2016 ▸). We restrict our analysis of this example to the two cells marked by A and B in Figs. 3 ▸(*a*)–3 ▸(*c*) as the corresponding fluorescence image [Fig. 3 ▸(*f*)] informs us that they are nicely spread out and attached to the substrate. The orientation and anisotropy maps in Figs. 3 ▸(*b*) and 3 ▸(*c*) clearly show that the cell outlines are more distinct when applying the optimal *q* range, since the cellular material is both anisotropic and oriented, whereas the background is not. By contrast, if the *q* range is increased, the signal is ‘diluted’ by featureless background signal. The histograms in Figs. 3 ▸(*d*) and 3 ▸(*e*) are based on the data shown in Figs. 3 ▸(*b*) and 3 ▸(*c*) and thus show the orientation and anisotropy distributions, respectively, of cells A and B. The orientation distribution [Fig. 3 ▸(*d*)] for the optimum range (Δ*q*_2opt_, red) shows dominant distribution features at ±90° (corresponding to the vertical direction in the image), while the one of the maximum range (Δ*q*_24_, cyan) shows a uniform distribution over all angles. In the case of the anisotropy [Fig. 3 ▸(*e*)], the distribution shifts towards the lower values when increasing the *q* range, compare cyan with red, in agreement with our qualitative assessment regarding Fig. 3 ▸(*c*). Taken together, these quantitative comparisons highlight the importance of choosing the optimal *q* range for detecting the weak cellular SAXS signal and support our method for determining *q*_opt_ as discussed above. Notably, the anisotropy is more affected by the *q* range than the orientation. The anisotropy is obtained by the circular variance of the azimuthal intensity profile, while the orientation is obtained from the circular mean. Increasing the *q* range beyond the *q*_opt_ introduces noise to the azimuthal intensity profile at all angles, and the extent of this noise grows with *q*. As the *q* range is increased, the circular mean remains stable until the noise level exceeds a certain threshold. However, the circular variance is directly affected by the added noise, and thus it changes promptly. To illustrate this argument, a simulation comparing the sensitivity of the circular mean and the circular variance on the noise level is shown in Fig. S9.

A closer comparison of the local orientation results of the optimum *q* range with the dark-field contrast image reveals a correlation between orientation and cell shape. Cell A is elongated in the vertical direction (±90°, purple). The right side of cell B is elongated in a predominantly horizontal direction (0°, green). The anisotropy is increased in certain cellular regions and high anisotropy values are found at the cell contours, whereas low values are found in the nuclear regions in both cells, coinciding with high electron density found in the dark-field image [Fig. 3 ▸(*a*)]. We thus hypothesize the oriented anisotropic signal to stem from cytoskeletal structures within the cytoplasm.

To investigate this hypothesis further, we compare a fluorescence micrograph [Fig. 3 ▸(*f*), inverted grayscale, dark regions show labeled keratin bundles (Weinhausen *et al.*, 2012 ▸)] of the example region with the SAXS data. We focus on two regions, 1 and 2 (black boxes), where the ordered keratin network is visible in the fluorescence image. Region 1 contains a thick keratin bundle forming a loop-like structure and this region is marked by the white outline in Fig. 3 ▸(*g*), left. The black lines overlaid to the dark-field image show the orientation and anisotropy by their angle and length, respectively, and we can follow the loop structure here as well. Region 2 shows a cellular extension that contains a thick keratin bundle and the SAXS data [Fig. 3 ▸(*g*), right] show increased electron density (dark-field image) and aligned anisotropic signal (black lines). More examples are shown in Figs. S10 and S11.

### 3.3. Radiation damage from repeated imaging of cells

**Figure 4:**
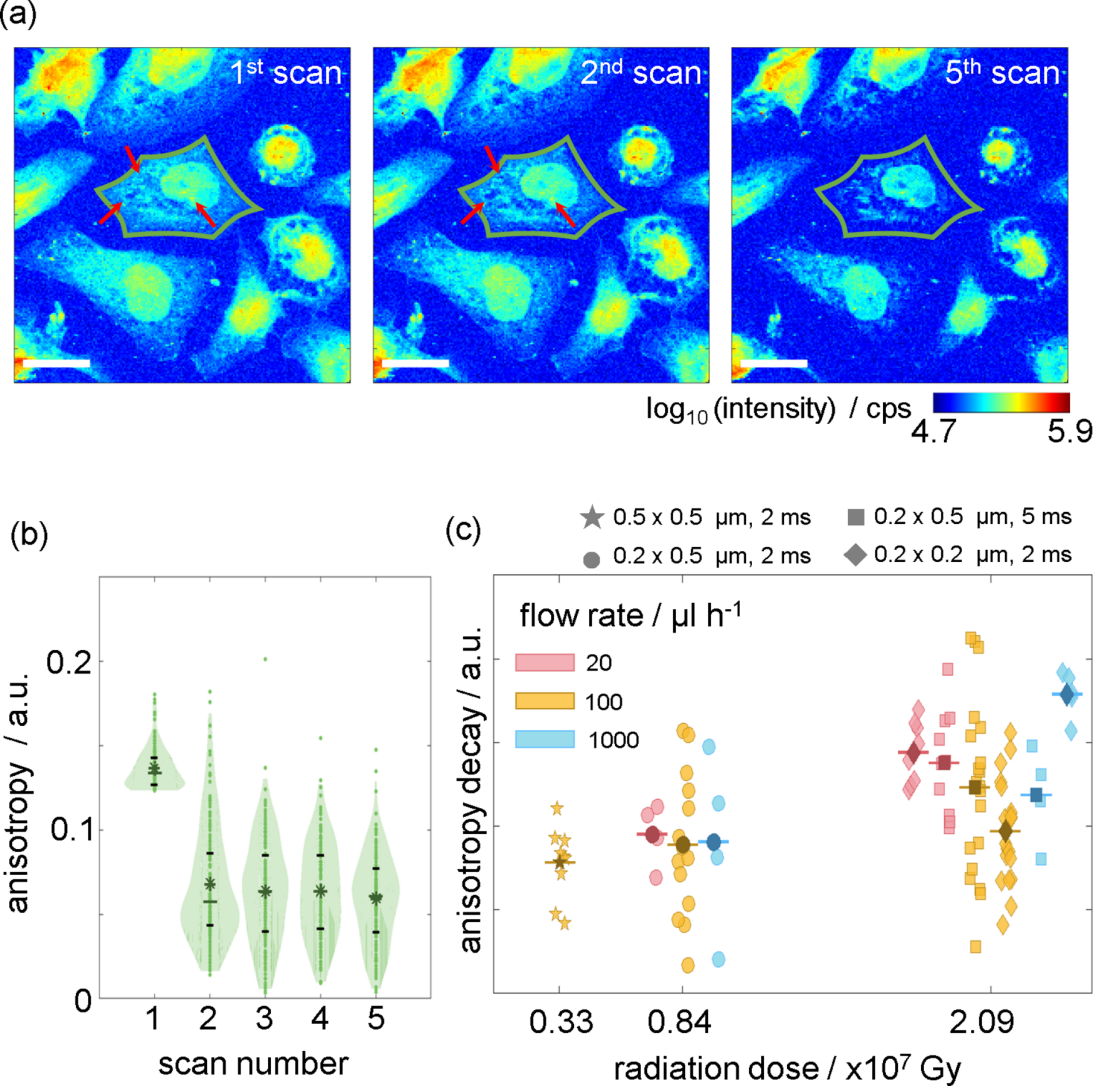
Analysis of radiation damage. (*a*) First (left), second (center) and fifth (right) dark-field contrast images obtained from multiple scans of the same region. Each cell in the first scan is labeled individually, represented by different colors in Fig. S7. Scale bars: 20 µm. (*b*) Anisotropy distribution of a single example cell that is labeled in green in panel (*a*) plotted against the scan number. In the cytoplasm region of individually labeled cells, while excluding the nuclear region, only pixels with anisotropy values greater than the 99th percentile of the anisotropy distribution of the background region are used. The horizontal lines in each violin plot represent the 25%, 50% and 75% quartiles from the bottom to the top, and the asterisks denote the means; the 50% quartile corresponds to the median. (*c*) Degree of anisotropy decay from the first to the second scan of individual cells using different measurement conditions (step size and exposure times, see legend above the figure). The color of each symbol corresponds to the specified buffer flow rate. Darker symbols show the mean values for each respective condition.

Cellular structures are generally very sensitive to the high radiation dose input by a focused X-ray beam. In order to investigate how this radiation damage influences the anisotropic oriented signal stemming from cytoskeletal structures, we scan the same sample region multiple times with the same scanning parameters, *i.e.* step size and exposure time. An example series of dark-field contrast images of the first, second and fifth scan of one such region is shown in Fig. 4 ▸(*a*). Visual inspection for comparison of the first and second dark-field image shows changes in the cytoplasmic regions, where the intensity values are slightly decreased, yet the cell shapes are still intact and clearly distinguished. However, the cell shapes are deteriorated in the fifth-scan image, indicating mass loss in the samples. In contrast, the nucleus appears more resistant to the radiation and the shapes of the nuclei are still clearly visible in the fifth scan. As the cellular material persist on the substrate after the first scan and even after four scans, we apply the analysis explained in Section 3.2 to these repeated scans. By this analysis, we are able to detect if, despite only limited mass loss that occurs during the first scan, intracellular components are compromised concerning the anisotropy they cause in the scattering signal. In Fig. 4 ▸(*b*), we show the anisotropy distributions of the single cell outlined in green in Fig. 4 ▸(*a*) plotted against the scan numbers. A strong decrease in anisotropy is visible only between the first and the second scan, thereafter we do not observe considerable changes anymore. This result indicates that most ordered structures are destroyed during the first scan already. This is remarkable, as the dark-field image calculated from the second scan [Fig. 4 ▸(*a*), center] shows only limited mass loss compared with the one from the first scan (left), with the cytoplasmic regions retained.

Considering the strong decrease in anisotropy after the first scan, we quantify the extent of radiation damage of the ordered structures using the anisotropy decay defined as the difference in mean value between the first and the second distribution. The anisotropy decay values of individual cells measured using different scanning step sizes and exposure times are plotted against the corresponding radiation dose, as shown in Fig. 4 ▸(*c*). Different scanning parameter sets are denoted by different symbols, and the flow rates are represented by different colors. One notable result is that as the radiation dose increases, the mean as well as the variance of anisotropy decay distributions increases. The large variance is due to the natural cell-to-cell variability. Given that anisotropy decay cannot be negative, the possible anisotropy decay value is equivalent to or smaller than the initial anisotropy values. It follows that some cells whose mean anisotropy values are initially small and which are completely damaged afterward result in small anisotropy decay values. Conversely, other cells initially showing large anisotropy values result in various values depending on the extent of the damage. A higher radiation dose yields a higher incidence of complete damage, producing a large variance in anisotropy decays. Consequently, a large variance in higher radiation dose explains more severe destruction of intracellular structures. Anisotropy decays with different extents of radiation damage are simulated in Fig. S12.

In contrast to the positive correlation between anisotropy decay and radiation dose, no systematic correlation is observed between the flow rates and the anisotropy decay. Further experiments will be necessary to investigate whether flow rates beyond the maximum one used here (1000 µL h^−1^, corresponding to 9.26 µm s^−1^) will diminish the radiation damage on the cell samples. Additionally, it will be interesting to investigate the influence of the scan direction (*i.e.* in parallel or perpendicular to the flow direction), thus testing if we are able to ‘outrun’ the diffusion of radicals to unexposed regions on the sample or not. The damage to cells from X-ray radiation is a result of both direct and indirect processes (Riley, 1994 ▸; Hémonnot & Köster, 2017 ▸; Nicolas *et al.*, 2019*a* ▸). The indirect processes are typically caused by free radicals generated by water radiolysis (Le Caër, 2011 ▸; Stark, 2005 ▸). These radicals react with cellular components, and with other radicals (Stark, 2005 ▸; Nordberg & Arnér, 2001 ▸; Kai *et al.*, 2025 ▸). In pure-water environments, these reactions occur extremely fast, ranging from picoseconds to nanoseconds (Le Caër, 2011 ▸; Loh *et al.*, 2020 ▸; Riley, 1994 ▸). Additionally, radicals are transported through the water very quickly due to the Grotthuss mechanism. Direct processes include ionization and breaking of chemical bonds (Leccia *et al.*, 2010 ▸; Weinhausen *et al.*, 2012 ▸; Nicolas *et al.*, 2019*a* ▸), which cause the cellular components to gradually lose their mass and can be monitored by intensity changes in the dark-field contrast image series, see Fig. 4 ▸(*a*). Regarding the anisotropy decay in Fig. 4 ▸(*b*), we speculate that direct processes may damage the intracellular structures, which give rise to a decay in the orientation and anisotropy signal.

## 4. Summary and conclusions

We demonstrate a method to derive quantitative information from fixed-hydrated biological cells using fast-scanning SAXS, despite short exposure times and low SNR. In order to maintain a hydrated state of the cells and ensure a close-to-physiological sample environment, a dedicated microfluidic chamber is employed. This chamber enables stable operation for prolonged periods of time of the order of several hours with a wide range of possible flow rates. In our study, we flush buffer through the chamber, because we study fixed-hydrated samples; however, it is equally well possible to replace the buffer by cell medium when investigating living cells. The collected SAXS data are analyzed using a novel noise-filtering method based on optimizing the *q* range such that the scattering angles with meaningful signal are captured but as little of the noisy signal is included. This approach allows for the reduction of background noise without any observable information loss, as observed from inspecting the dark-field contrast images. The optimized *q* range also enables us to derive orientation and anisotropy of sub-cellular structures using the circular mean and variance of the azimuthal intensity profile. Comparing this signal with visible-light fluorescence images of the keratin bundles and networks in the cells, we observe a strong correlation, confirming that structural information collected using fast-scanning SAXS indeed originates from intracellular structures. Biological cells are very sensitive to radiation damage. Therefore, in order to demonstrate the utility of our approach combining microfluidics and dedicated data analysis for fast-scanning SAXS, we perform systematic analyses of radiation-induced damage to intracellular structures. Interestingly, damage to the structures associated with anisotropy occurs already during the first scan, despite the very short exposure time of a few milliseconds. Thereafter, the anisotropy signal remains at a low level but does not decay further. Notably, this damage is hardly observed in the dark-field contrast images. As expected, the extent of the radiation damage is positively correlated with the radiation dose. However, we do not observe a correlation with the flow rate in the range used in our experiments. Nevertheless, it is worthwhile to investigate ways to reduce the dose imposed by the X-rays on the cells, in particular with regard to possible applications to living cells (Weinhausen *et al.*, 2014 ▸). This can in principle be achieved by using shorter exposure times or lower photon flux. However, the challenge of an even poorer SNR will have to be addressed. An increase in step size would also decrease the dose, at the expense of a lower lateral resolution in real space. In the current study, we carefully chose the exposure time, photon flux and step size to minimize the dose as much as possible while still being able to quantitatively analyze the obtained signal.

Our study demonstrates that state-of-the-art synchrotron radiation with high flux and focused beams allows for the analysis of structural information stemming from intracellular structures, beyond mere cell-shape segmentation. Our approach, including the X-ray compatible microfluidic chamber, can be extended to living cells in a straightforward manner. It will be highly interesting to see if and how the chemical fixation changes orientation and alignment in comparison with living unfixed cells. Moreover, the method is applicable to other cell types whose internal structures show a pronounced degree of order, such as actin bundles in stereocilia (Piazza *et al.*, 2014 ▸), actin stress fibers in cardiomyocytes (Bernhardt *et al.*, 2016 ▸, 2017 ▸; Reichardt *et al.*, 2020 ▸) and in cardiac tissue (Nicolas *et al.*, 2019*a* ▸,*b* ▸).

## Supplementary Material

Figures S1 to S12. DOI: 10.1107/S1600577526002018/vl5055sup1.pdf

## Figures and Tables

**Table 1:** Measurement parameters and calculated radiation doses

Δ*y* (µm)	Δ*z* (µm)	Exposure time (ms)	Dose (10^7^ Gy)
0.2	0.2	2	2.09
0.2	0.5	2	0.84
0.2	0.5	5	2.09
0.5	0.5	2	0.33
0.5	0.5	5	0.84

## Data Availability

The data included in this paper are uploaded on GRO.data and available at https://doi.org/10.25625/6CE04V.
