# Supplementary material for: Fast-scanning small-angle X-ray scattering of hydrated biological cells
Source: J Synchrotron Radiat. 2026 Mar 30;33(Pt 3):777–87. doi: 10.1107/S1600577526002018 (PMC13148594; doi:10.1107/S1600577526002018)
Supplement: Supplementary file 1 [file s-33-00777-sup1.pdf]

JOURNAL OF SYNCHROTRON RADIATION

Volume 33 (2026)

Supporting information for article:

Fast-scanning small-angle X-ray scattering of hydrated biological cells

Boram Yu, Mangalika Sinha, Rita Mendes Da Silva, Ulrike Rölleke, Manfred Burghammer and Sarah Köster

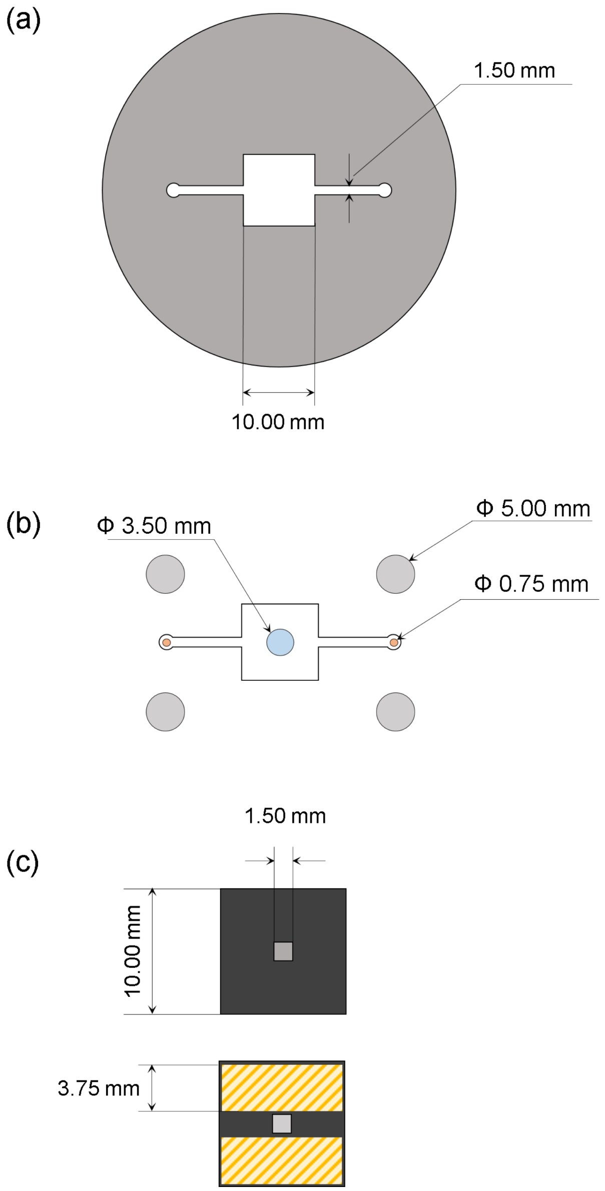

Figure S1: (a) Photomask design. The outer diameter is 2 inches and the structure is positioned in the center. (b) Structure casted in PDMS with punched holes; diameters: 0.75 mm for the inlet and outlet (orange), 3.5 mm for the X-ray observation window (blue), and 5.0 mm for the screws (gray). (c) SiN membranes; the yellow-hatched area on the bottom membrane is the custom-ordered spacer. The thickness of the silicon frame is 200 μm and the thickness of the SiN window is 1 μm. The spacer thickness is 20 μm.

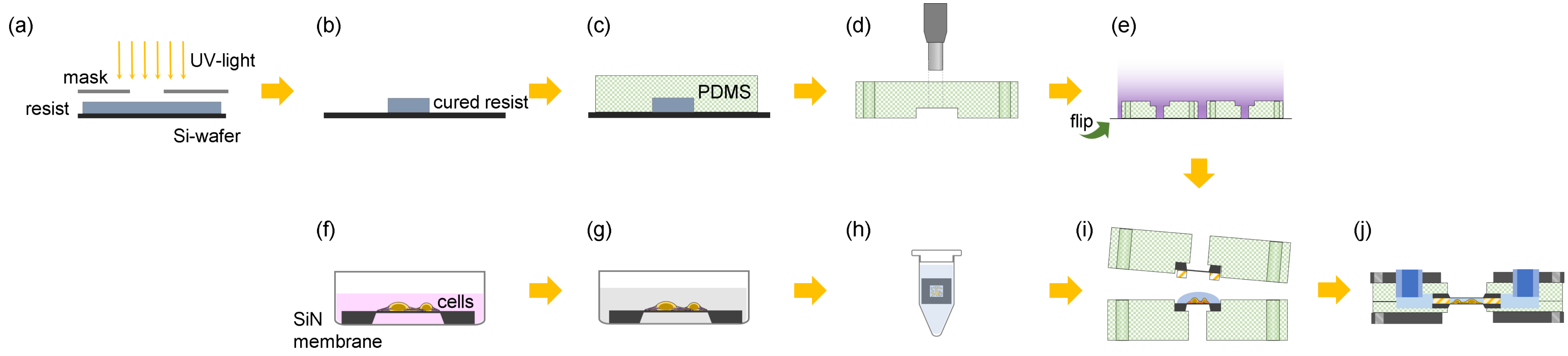

Figure S2: Chamber fabrication process; a to d: photolithography and PDMS replication; f to h: sample preparation process as described in Sec. 2.1 of the main text. (a) A thin layer of resist is spin coated on a Si-wafer and exposed to UV-light through the photomask. (b) After developing, the UV-exposed region in the resist remains and forms the channel structure. (c) PDMS block is cast from the master. (d) Access holes for X-rays, screws, and tubing are punched. (e) Plasma-activation of the surface of the two PDMS blocks. (f) Cells are grown on SiN membranes. (g) Cells are chemically fixed by 4 % formaldehyde and stored in PBS. (h) SiN membrane with cells is stored in PBS. (i) SiN membranes with cells and with SU-8 spacer are placed on each PDMS blocks and sandwiched. (j) Tubings are placed and the metal frames clamp the entire device with screws.

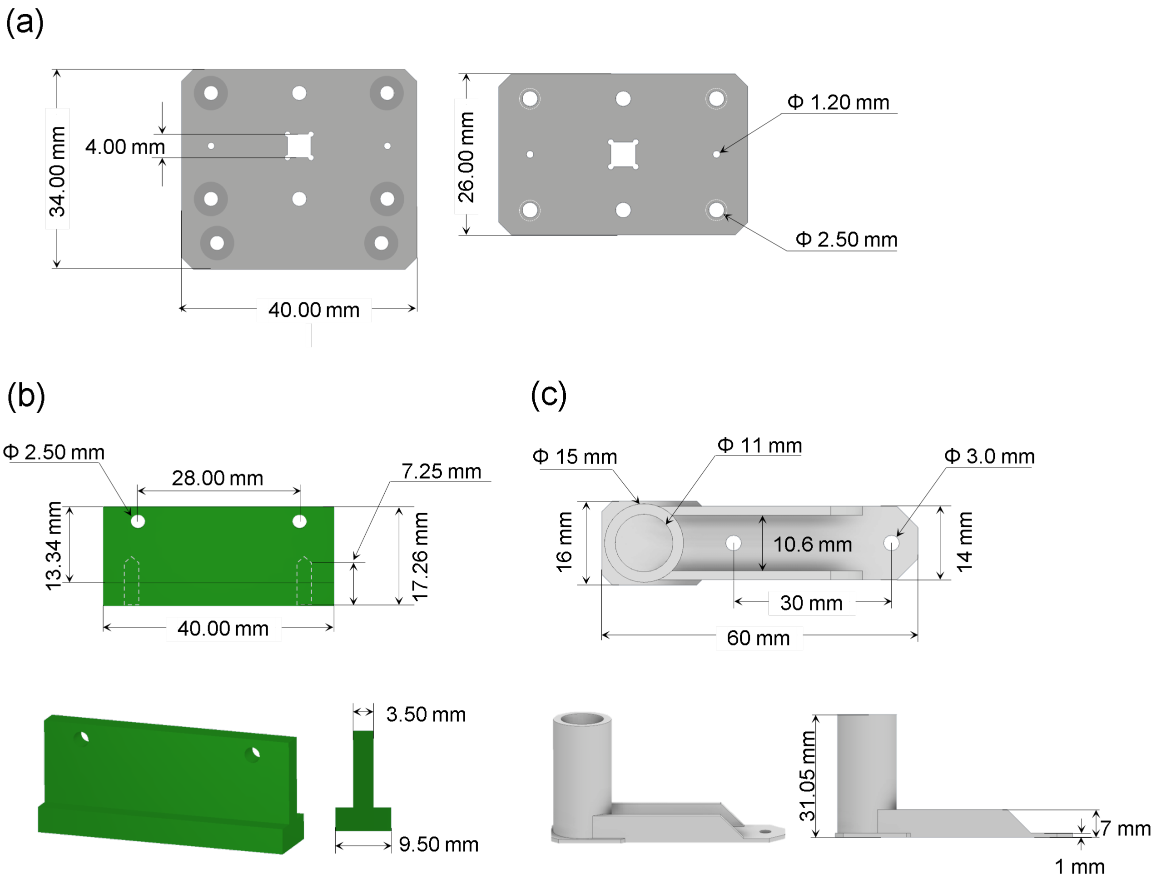

Figure S3: Design of the metal frames and the chamber stand. (a) Metal frames; left: located downstream with an extension to attach it to the chamber stand in b; right: located upstream; holes with a diameter of 2.5 mm are included for clamping using screws; the square holes in the center of both frames constitute the X-ray observation windows. The weight of two frames including the four screws is 17.2 g. To avoid collisions with the heads of the screws, the screw holes in the larger metal plate (shown on the left) are tapered (darker gray region). The screw holes in the the smaller metal plate (shown on the right) contain threads. (b) Design of the chamber stand; the two holes on top are used to attach this part to the metal frames shown in a. The thickness of the stand is adequately selected to load the PDMS on it for better stability. The dashed lines illustrate embedded screw holes, which are used for mounting on the sample stage. The weight of the stand is 22.1 g. (c) The design of the waste container is compatible with 1.5 mL or 2 mL reaction tubes. The chamber stand in b is located between the extended wing structures at the bottom. The adapter plate for the piezo sample stage is combined with the stand by two screws, which pass through the waste container. After assembly of all components, the final weight of the entire chamber apertures is approximately 70 g, depending on the exact weight of the PDMS parts.

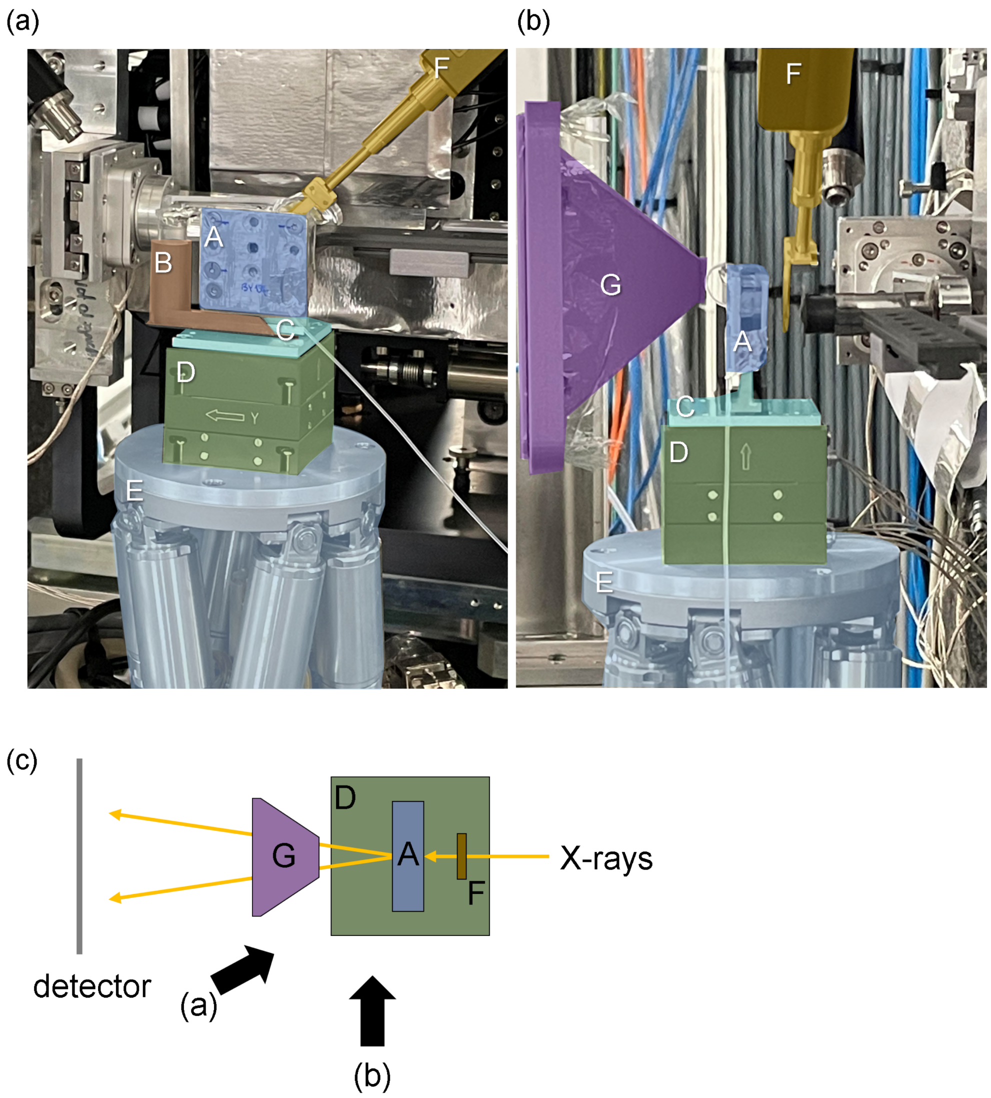

Figure S4: Photographs (a, b) and schematic (c) of the end station with the mounted sample. **A**: sample chamber, visible are the metal frames; **B**: waste container; **C**: chamber stand; **D**: piezo sample stage; **E**: hexapod; **F**: X-ray aperture and holder; **G**: flight tube. (a) and (b) Photographs taken form different angles; note that the picture in panel a was taken without the flight tube. (c) Schematic top view; black arrows indicate the observation angles for a and b.

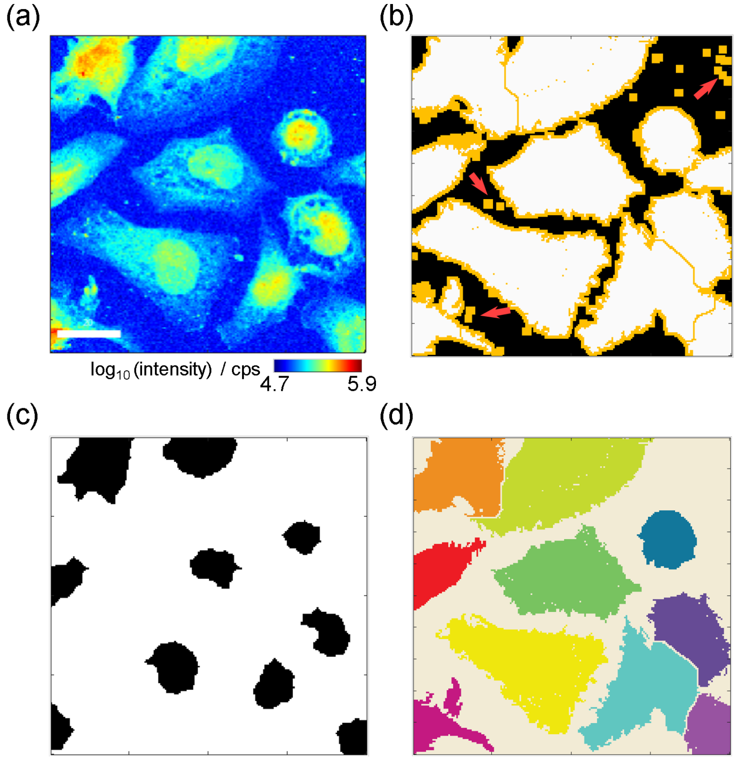

Figure S5: Example of a dark field contrast image and masks generated by different methods. (a) Original dark field contrast image. (b) Background and foreground mask generated by ACSAT. The white regions indicate foreground, and the black regions indicate the background. Added pixels after applying a Gaussian filter are indicated by red arrows. The yellow regions indicate gaps between the background and the foreground after morphological dilation with a circular structure element with a diameter of 3 pixels. (c) Nuclear mask generated by morphological reconstruction. (d) Final cell annotation after applying the marker-controlled watershed method.

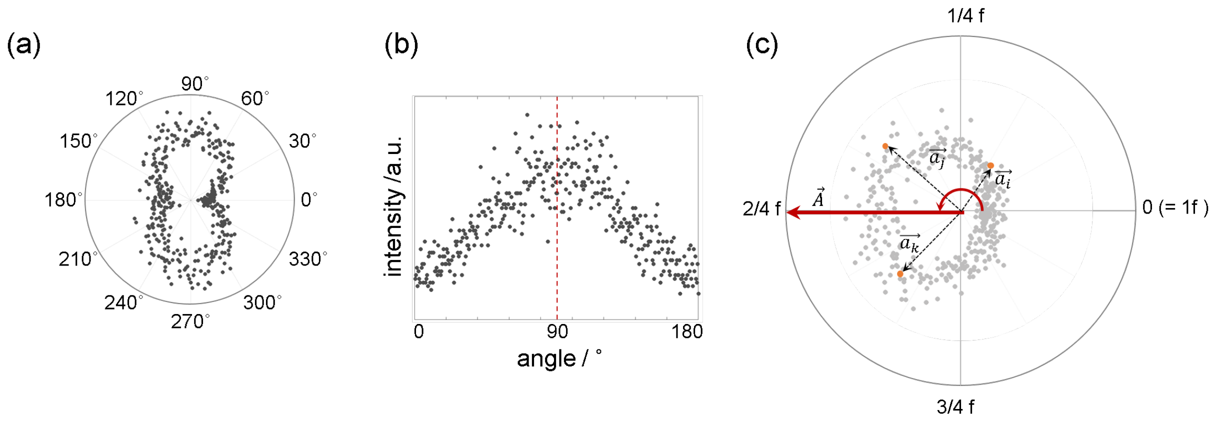

Figure S6: Graphical interpretation of the circular mean and variance calculation. (a) Azimuthal intensity profile of an example SAXS scattering pattern represented over the full range of angles from 0 to 360°. (b) As the azimuthal intensity profile is symmetric with a periodicity of 180°, the azimuthal intensity profile can be averaged and plotted over a range of 0 to 180°. The red dashed line indicates 90°. (c) In order to obtain the orientation from the circular mean and the anisotropy from the circular variance, we plot each data point in a circular manner, where one revolution represents one cycle frequency $f = 180°$. The vector $\vec{a_i}$ points from the center to a specific data point. For illustration, three example data points are marked in orange, and the corresponding vectors are shown as dashed arrows, $\vec{a_i}$, $\vec{a_j}$, and $\vec{a_k}$. The vector $\vec{A}$ is the sum of all vectors $\vec{a_i}$. The circular mean is the angle of $\vec{A}$ with respect to the horizontal direction, and the anisotropy is the magnitude of $\vec{A}$, which is zero in case the data points form a perfect circle, i.e., the intensity is equally distributed over all angles. The orientation of the scattering pattern is parallel to $\vec{A}$.

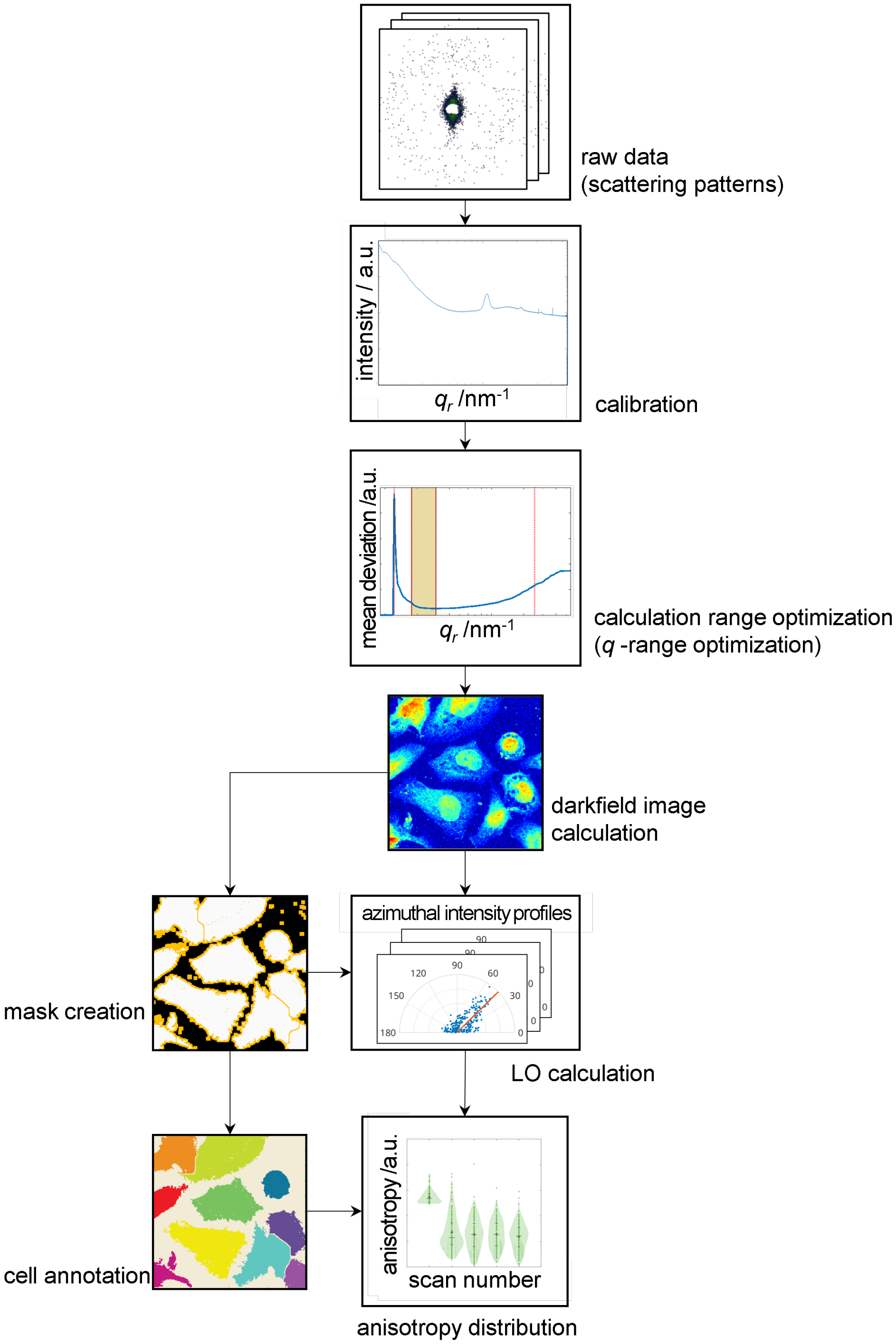

Figure S7: Work flow for the data analysis (further details are described in Sec. 2.4 in the main text): Calibration is performed using AgBeh dataset; the q-range is optimized to minimize the noise using a selected dataset; a dark field image is calculated; the local orientation is determined, including orientation and anisotropy maps; to analyze the anisotropy decays of single cells, cells in a single dark field image are segmented and annotated; the anisotropy distribution of a single cell is represented as one violin plot in the figure.

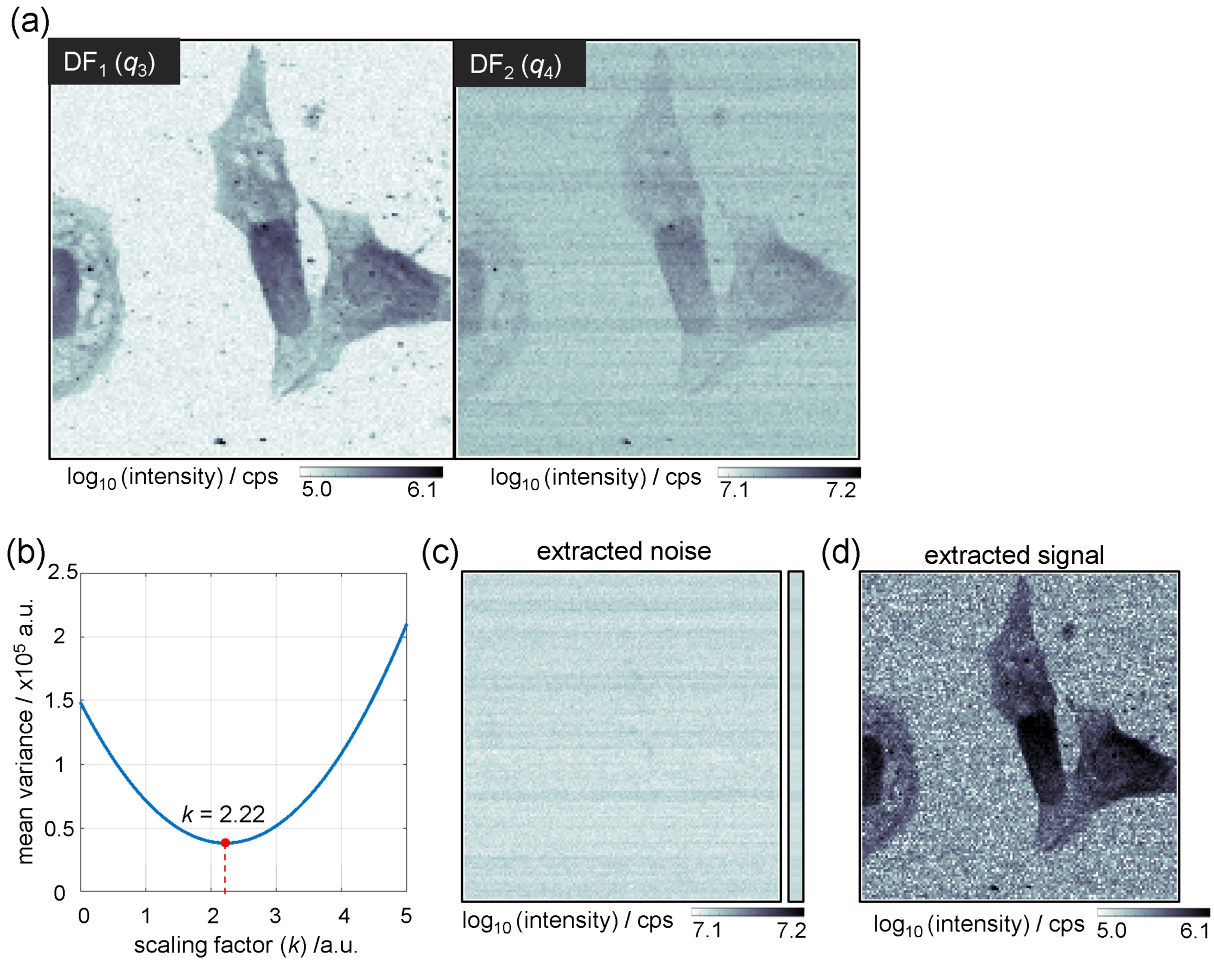

Figure S8: Noise extraction and analysis. (a) Dark field contrast images: The stripe-shaped noise is minimized in DF_1, and maximized in DF_2, as described in Sec. 3.1 in the main text. Assuming the stripe noise is additive, the cell shapes in DF_2 can be considered as a linearly scaled version of DF_1 with a scaling parameter of k. (b) The mean variance of differences between pixel values in DF_2 and $k \times DF_1$ is plotted against k; k is determined by the minimum of the curve. (c) DF_2 - $k \times DF_1$. Only the stripe pattern is extracted, showing that the noise is indeed additive. The longitudinal box on the right hand side shows the averaged intensity of each row. (d) Noise-subtracted DF_2: Averaged intensities are subtracted row by row from DF_2. The cell shapes are still visible.

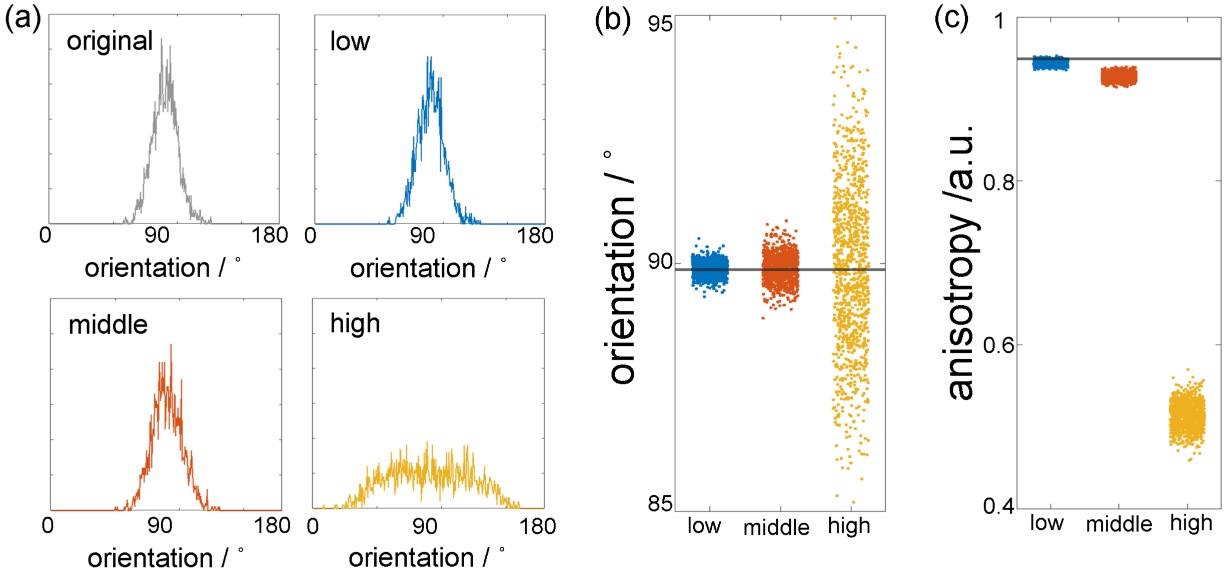

Figure S9: Simulation showing the difference in sensitivity between the orientation and anisotropy. (a) The input signal is a merged data set of two random datasets, each consisting of 1,000 random normally distribution numbers with a mean of 90° or 270°, respectively, and a standard deviation of 10° to obtain a simulated data set similar to the real data. The added noise consists of random integer sets, uniformly distributed in a range of [-5, 5], [-10, 10], and [-50, 50], denoted 'low', 'middle', and 'high', respectively. (b) Corresponding orientation and (c) anisotropy values after repeating the simulation 1000 times. The black lines show the values of the original signal. In the azimuthal intensity profile (panel a), the position of the peak determines the orientation values, whereas the height of the peak determines the anisotropy. Since a higher noise level reduces the height of the peak, but does not change its position, the orientation values are distributed around the original dataset's value (90°) with different variances in the noise level, whereas the anisotropy values are shifted towards lower values with larger variance for higher noise.

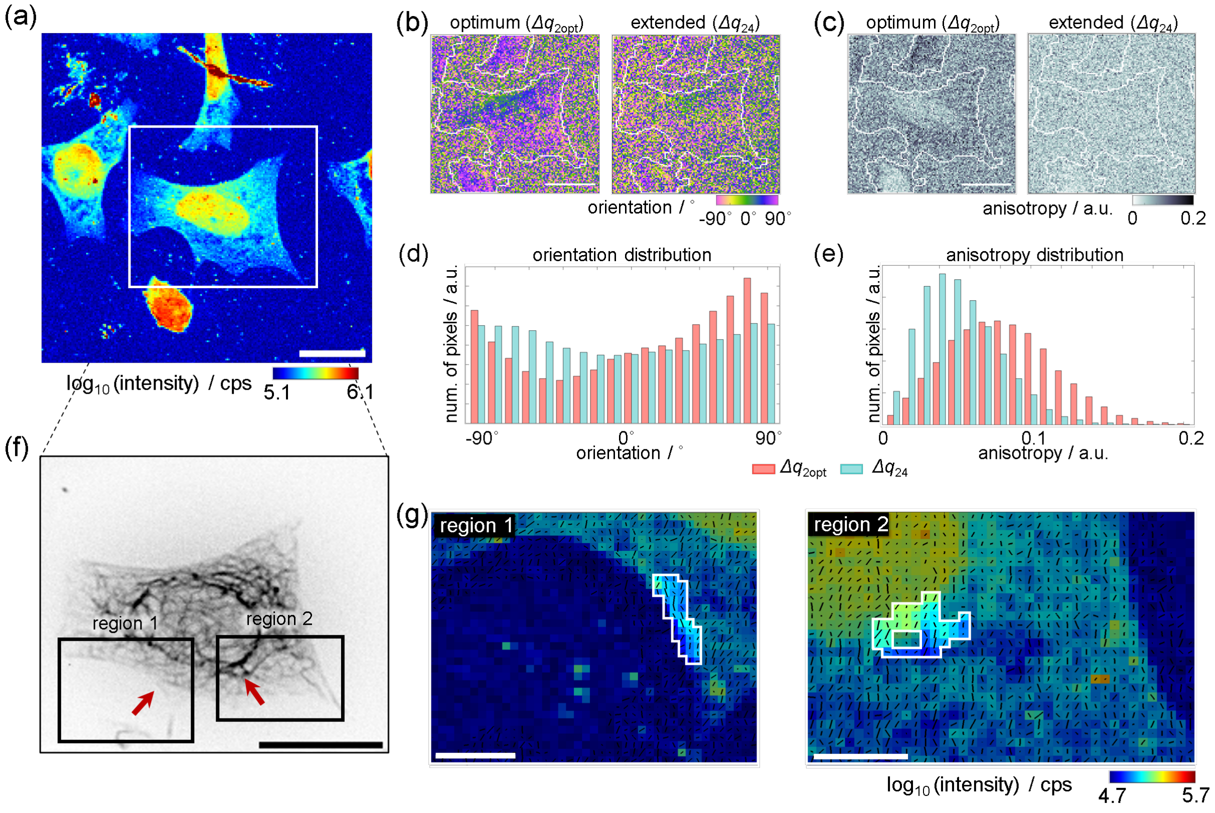

Figure S10: Local orientation analysis (see Fig. 3 in the main text) of an additional data set; the step size is 0.5×0.5 μm and the exposure time is 2 ms. (a) Dark field contrast image calculated using the optimized q-range. (b) Orientation maps of the white rectangular region in panel a, calculated using different q-ranges: $\Delta q_{2\text{opt}}$ from q_2 to q_{opt} and Δq_{24} from q_2 to q_4. (c) Corresponding anisotropy maps. The white outlines in panel b and c delineate the border of the background area used for the background subtraction. Histograms of (d) orientation and (e) anisotropy distributions of the cell located in the center of panels a-c. (f) Inverted gray scale epifluorescence image of the same cell, acquired prior to the microfluidic chamber assembly. The dark structures are distinct keratin bundles within the cell. (g) Dark field contrast images of the two regions 1 and 2 within the black boxes in panel f, where strongly ordered keratin structures are visible. The black lines in each pixel represent the orientation and anisotropy by their angle and length, respectively. Scale bars in panel a and f: 20 μm, in panel g: 5 μm.

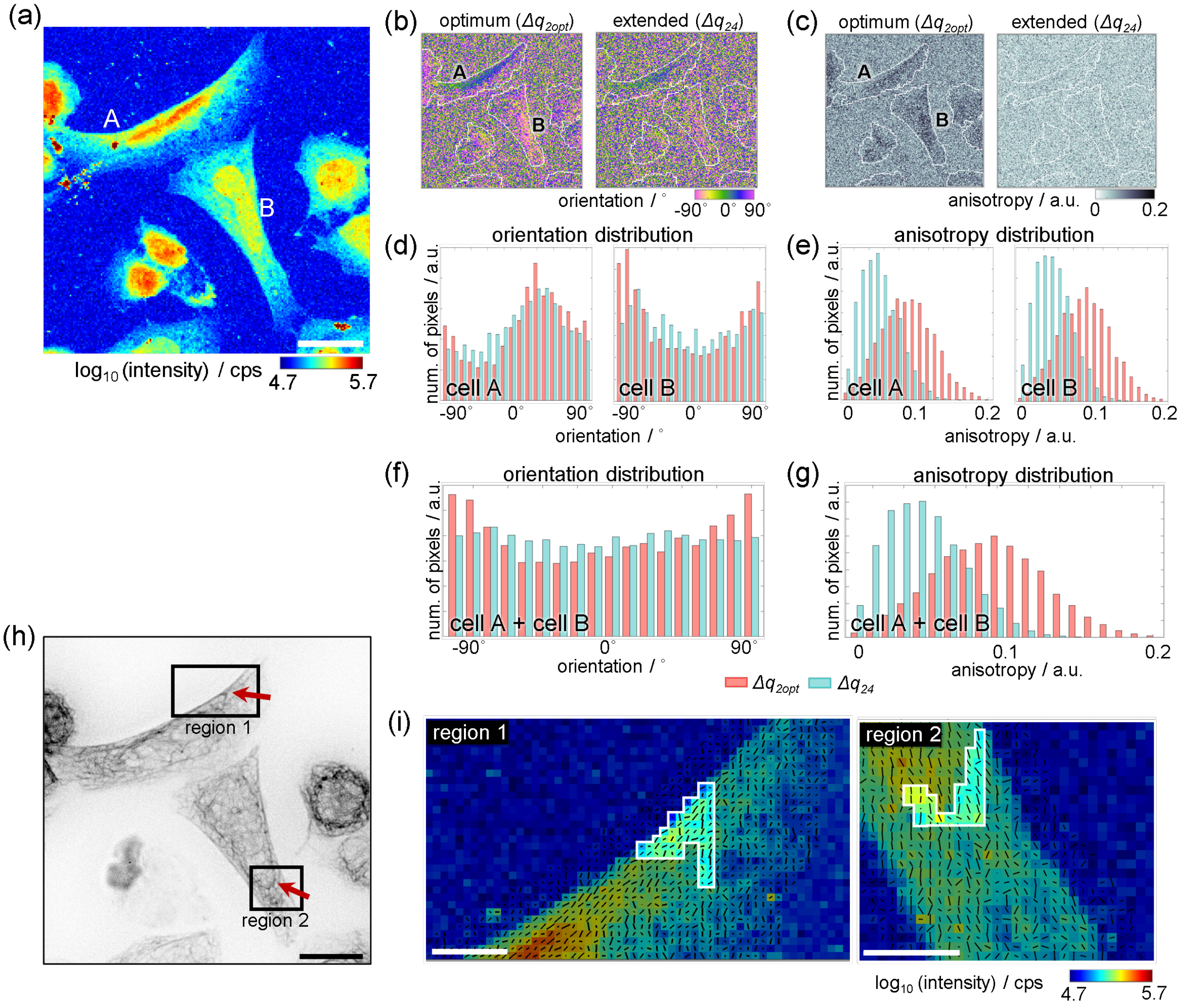

Figure S11: Local orientation analysis (see Fig. 3 in the main text) of an additional data set; the step size is 0.5×0.5 μm and the exposure time is 2 ms. (a) Dark field contrast image calculated using the optimized q-range. (b) Orientation maps of the region shown in panel a, calculated using different q-ranges: $\Delta q_{2\mathrm{opt}}$ from q_2 to q_{opt} and Δq_{24} from q_2 to q_4. (c) Corresponding anisotropy maps. The white outlines in panel b and c delineate the border of the background area used for the background subtraction. Histograms of (d,f) orientation and (e,g) anisotropy distributions of the cells A and B indicated in panels a-c. (h) Inverted gray scale epifluorescence image of the same cells, acquired prior to the microfluidic chamber assembly. The dark structures are distinct keratin bundles within the cells. (i) Dark field contrast images of the two regions 1 and 2 within the black boxes in panel h, where strongly ordered keratin structures are visible. The black lines in each pixel represent the orientation and anisotropy by their angle and length, respectively. Scale bars in panel a and h: 20 μm, in panel i: 5 μm.

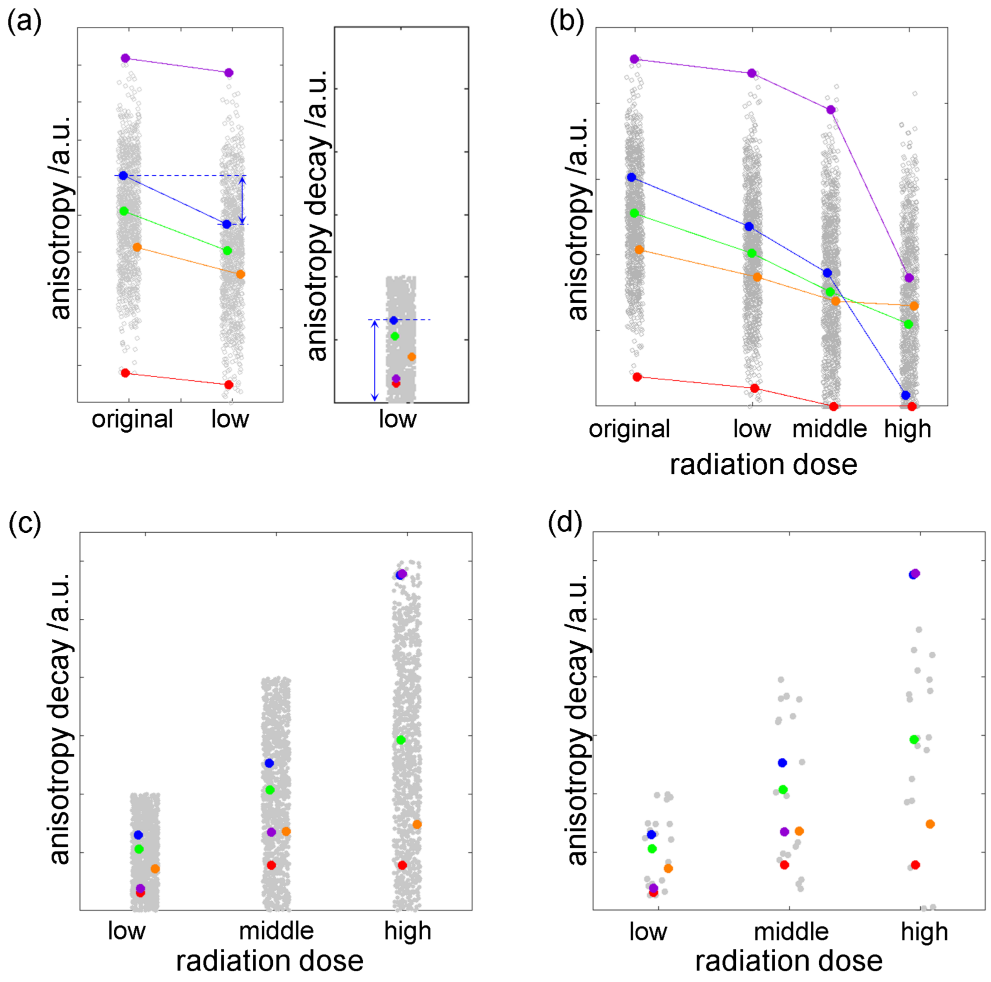

Figure S12: Simulation of anisotropy decay distribution. (a) Explanation of how to determine the anisotropy decay. The original signal consists of 1,000 random normally distributed numbers between [0, 1] with a mean of 0.5 and a standard deviation of 0.1. Each data point indicates an averaged anisotropy value of the cytoplasm region of a single cell; thus the 1,000 data points represent 1,000 cells. The anisotropy decay is the difference between the original and radiation-damaged anisotropy values, where the extent of the radiation damage is randomly selected within a constrained range according to the radiation dose (see blue dashed lines and arrow for an example). Any anisotropy decays smaller than zero are set to zero. In the visualization, five example data points (colored) are randomly selected and tracked. The lengths of the arrows are different in the left and the right plots due to the scaling. (b) Influence of different levels of radiation dose. The extent of radiation damage is set to [0, 0.2] for the low dose, [0, 0.4] for the middle dose, and [0, 0.6] for the high dose. (c) Anisotropy decay values of all 1,000 data points. The higher the dose the larger the variance. (d) 25 random data points, including the previously selected 5 data points, which exhibit a similar trend to Fig. 4 in the main text. This similarity explains that the large variance in the anisotropy decay at high doses indicates more damage.
